# Exercise Regulates the Metabolic Homeostasis of Methamphetamine Dependence

**DOI:** 10.3390/metabo12070606

**Published:** 2022-06-29

**Authors:** Xue Li, Kefeng Li, Zhicheng Zhu, Yu Jin, Zhanle Gao, Jisheng Xu, Li Zhang

**Affiliations:** 1 School of Sports Medicine and Health, Chengdu Sport University, Chengdu 610041, China; zhuc996@gmail.com (Z.Z.); jiny_1104@foxmail.com (Y.J.); gaozhanle@foxmail.com (Z.G.); xujisheng@cdsu.edu.cn (J.X.); 2 Department of Medicine, Quzhou College of Technology, Quzhou 324000, China; jmlikefeng@qzct.edu.cn; 3 Key Laboratory of Central CNS Regeneration (Ministry of Education), Guangdong-Hong Kong-Macau Institute of CNS Regeneration, Jinan University, Guangzhou 510632, China; zhangli_sd@hotmail.com

**Keywords:** metabolomics, drug addiction, methamphetamine, physical activity, GABA

## Abstract

Physical exercise is effective in enhancing cognitive function, reducing anxiety and depressive symptoms, reducing cravings, and improving quality of life in methamphetamine (METH) addiction. However, little is known about the effect of exercise on metabolic profiles. We performed LC/MS-based targeted metabolic profiling on serum samples to investigate the metabolic characteristics of METH dependence and find the differences between METH-dependent individuals and nonusers and evaluated the metabolomic profiles of individuals with METH dependence following aerobic exercise training. We identified a total of 201 metabolites, among which 115 were differentially expressed under METH use. Among the differentially regulated metabolites, 72 were selected as potential biomarkers. Further analysis identified 19 pathways, among which glyoxylate and dicarboxylate metabolism; alanine, aspartate, and glutamate metabolism; and citrate cycle were most significantly affected by METH. The aerobic exercise intervention differentially regulated 55 metabolites, of which 51 were selected as potential biomarkers and were mainly enriched in 10 pathways. Interestingly, alanine, aspartate, and glutamate metabolism and nitrogen metabolism were the remarkably affected pathways. Furthermore, METH increased the serum levels of glutamate and decreased GABA, whereas exercise decreased the serum levels of glutamate and increased GABA. Results suggested that METH dependency disturbed normal metabolic homeostasis, whereas exercise restored metabolism.

## 1. Introduction

Drug addiction is a chronic and recurrent brain disease that is characterized by compulsive drug craving, seeking, and continuous reckless use [1]. The occurrence and development of drug addiction and relapse involve multiple neural networks in the brain, including the reward, antireward/stress, and central immune systems [2]. The toxicity of addictive drugs drives the dysfunction and death of central nervous system cells, disrupts the intricate homeostasis of the nervous system, and results in a persistent neurochemical disturbance; furthermore, drug addiction increases the risk of psychological complications, cardiac lesions, and liver and lung diseases [3,4,5]. Although the etiology and pathogenesis of this illness have not yet been fully elucidated, a growing body of evidence suggests that addiction can be attributed to a persistent neurochemical disturbance that is initiated through a disruption in metabolism and could lead to different types of psychological disorders [6].

The perturbations of some neurotransmitter systems, including the norepinephrine, serotonin, and dopamine systems, have been found to be associated with drug addiction. Among these systems, the dopamine system is closely related to reward, reinforcement, and cravings in drug addiction. Methamphetamine (METH) is the primary amphetamine-type stimulant with high abuse potential and considerable neuropsychiatric toxicity. METH can disrupt the structure and function of the blood–brain barrier by modulating the connections between endothelial cells, inducing fluid phase transmigration, and neuroinflammation [7]. In addition, METH is highly lipid soluble, so it can rapidly penetrate into the brain [8]. With a structure similar to dopamine, METH can disrupt dopaminergic pathways and increase the release of glutamate in the cortex; the damage inflicted by excess cortical glutamate on GABAergic interneurons leads to the dysregulation of cortical signals [9]. In addition, oxidative stress, neuroinflammation, and apoptosis play an important role in METH-induced neurotoxicity [10]. A GC/MS metabolomics study using male Sprague–Dawley rats found that METH abuse elevated energy metabolism, accelerated the tricarboxylic acid (TCA) cycle and lipid metabolism, increased the levels of the excitatory amino acids glutamate and aspartate, and decreased alanine and glycine levels in serum [11]. The results of a study based on ultraperformance liquid chromatography with high-resolution time-of-flight mass spectrometry revealed changes in the levels of certain endogenous metabolites in the blood samples of illicit 3,4-methylenedioxymethamphetamine drug users when compared with those of nonusers; these changes could be related to an increased energy demand, serotonergic syndrome, and drug-induced neurotoxicity [12]. Accordingly, emerging metabolomics studies have revealed that the toxic effects of METH and other addictive drugs are linked to the disorder of some metabolites and related signaling pathways, and their exploration will help reveal the pathological process of drug addiction, withdrawal, and relapse.

Exercise has been identified as a cost-effective lifestyle intervention for the prevention and treatment of many metabolic diseases, such as obesity, type 2 diabetes, and hypertension [13]. Also, in drug addiction, exercise has shown good rehabilitation benefits and safety effects in assisting detoxification, alleviating withdrawal syndrome, inhibiting relapse, and improving brain function and cognitive level; therefore, exercise intervention is a potential auxiliary or even independent rehabilitation means especially in the context of the absence of safe and effective medications (FDA-approved) for the treatment of drug addiction [14,15,16,17]. For example, in the initial and maintenance stage of drug addiction, moderate-intensity exercise for more than 6 weeks can effectively reduce drug intake; in the withdrawal stage, moderate-intensity exercise for more than 12 weeks can significantly reduce drug-seeking behavior [18,19]. Similarly, Wang et al. found that METH cravings after acute aerobic exercise markedly reduced compared with those in the pre-exercise stage and that acute aerobic exercise facilitated the inhibitory performance of METH-dependent individuals [20]. Prior voluntary exercise can ameliorate METH-induced damages to dopaminergic and serotoninergic fibers [21]. Exercise training is supposed to prevent or suppress addiction behaviors; modulate several neural networks, including the dopaminergic reward system; and relieve the neural dysfunctions associated with addiction [22].

On the basis of the abovementioned studies, we speculate that exercise might reprogram metabolic homeostasis in METH dependence. Here, we investigated the metabolomic differences between METH-dependent participants and nonusers and compared the differential metabolic profiles of the exercise group and nonexercise group of METH-dependent individuals. We provided a snapshot of metabolic responses to exercise to help understand how these patterns explain the mechanisms underlying the widespread benefits of exercise in METH dependence.

## 2. Results

### 2.1. Alterations in Metabolic Profiles Driven by METH

**Figure 1:**
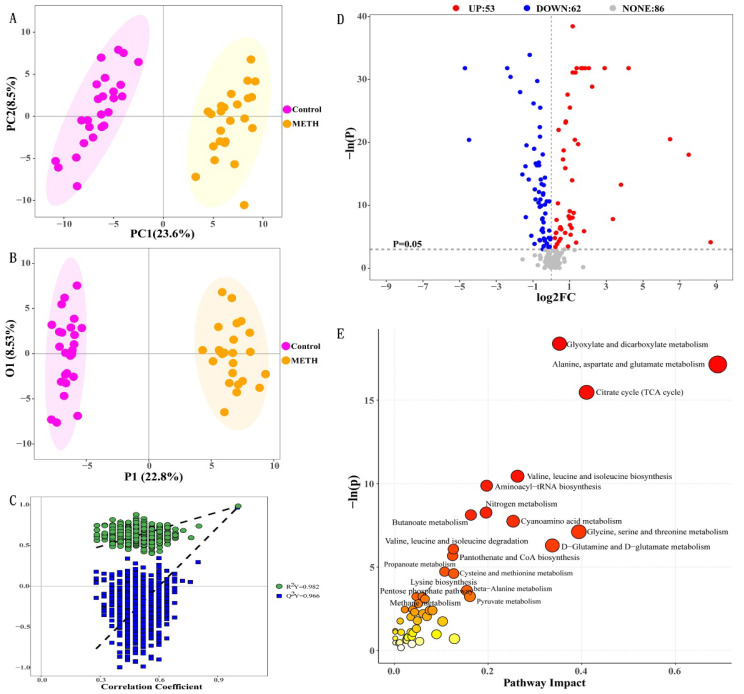
Differential metabolic profiles between the METH and control groups. (**A**) PCA score plot data of healthy controls (pink) versus METH dependents (yellow), (**B**) OPLS-DA scores plot data of healthy controls (pink) versus METH dependents (yellow), (**C**) permutation plot of the OPLS-DA models, (**D**) volcano plot of 115 significantly altered metabolites (*p* < 0.05), and (**E**) bubble plot of altered metabolic pathways analysis obtained via KEGG analysis and the comparison of METH with control groups.

We performed LC/MS-based targeted metabolic profiling on serum samples to investigate the metabolic characteristics of METH dependence and find the differences between METH-dependent individuals (METH group) and nonusers (control group). We detected 201 metabolites (For metabolite levels in serum for each group, please refer to Supplementary Material Table S1). Distinct metabolic profiles were observed between the METH and control groups. We first used an unsupervised multivariate statistical analysis method, namely, PCA, to identify overall metabolic differences between groups and the variation within the samples in each group (Figure 1A). The PCA results clearly suggested distinctions between the metabolic profiles of healthy people and METH abusers. A supervised analysis method, OPLS-DA was then performed and accounted for class discrimination with increased clarity (Figure 1B). The cumulative R2Y and Q2Y of the OPLS-DA score plot were 0.982 and 0.966, respectively (Figure 1C). Moreover, the intercept for the Q2 regression line was negative. These results indicated that the validation plots could ensure the reliability of the established OPLS-DA models. As shown in Figure 1B, METH dependents and nonusers were clearly divided into two clusters. This clustering pattern implied that serum metabolic patterns significantly changed with METH use.

On the basis of the successful differentiation between the METH group and the control group, we began to search for potential metabolites that might result in differences between groups. A volcano plot is helpful for selecting differential (statistically significantly changed) metabolites. In accordance with the threshold value (*p* < 0.05), 115 qualified differentially regulated metabolites were selected (Table 1). A total of 53 differential metabolites (red dots) in the right top corner were increased and 62 differential metabolites (blue dots) in the left top corner were decreased in the METH group (Figure 1D) relative to the control. Of these differential metabolites, 72 were selected as potential biomarkers with VIP > 1 (Table 1, top 72). Enrichment analysis was performed on the 72 differentially expressed metabolites via the KEGG database to elucidate the mechanism of metabolic pathway changes in METH dependence. As shown in Figure 1E, which presents all molecular pathways with P values less than 0.05, the differentially regulated metabolites were mainly enriched in 19 pathways, including glyoxylate and dicarboxylate metabolism; alanine, aspartate, and glutamate metabolism; citrate cycle (TCA cycle); valine, leucine, and isoleucine biosynthesis; aminoacyl–tRNA biosynthesis; nitrogen metabolism; butanoate metabolism; cyanoamino acid metabolism; glycine, serine, and threonine metabolism; valine, leucine, and isoleucine degradation; D-glutamine and D-glutamate metabolism; pantothenate and CoA biosynthesis; propanoate metabolism; cysteine and methionine metabolism; lysine biosynthesis; beta-alanine metabolism; pentose phosphate pathway; methane metabolism; and pyruvate metabolism.

### 2.2. Changes in the Metabolite Profiles of METH Abusers after Exercise Intervention

**Figure 2:**
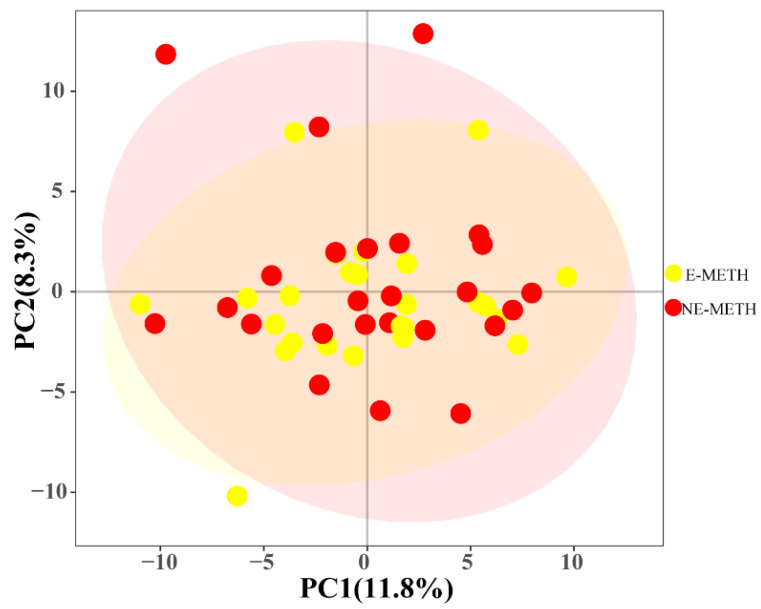
PCA score plot data of the NE-METH group versus the E-METH group.

The above experiment showed that METH could significantly change the metabolite profiles of individuals with METH dependence. We next investigated what happens to the metabolite profiles of individuals with METH dependence following aerobic exercise training. A total of 50 males with METH dependence were selected from a rehabilitation center and randomly divided into two groups. One group performed normal activities at the drug treatment center (NE-METH group), and the other group received regular aerobic exercise for 48 weeks (E-METH group). After the exercise intervention, blood samples were taken for metabolomic analysis. We analyzed the metabolite profiles of the two groups before exercise intervention and found no significant differences in their serum metabolite profiles (Figure 2).

**Figure 3:**
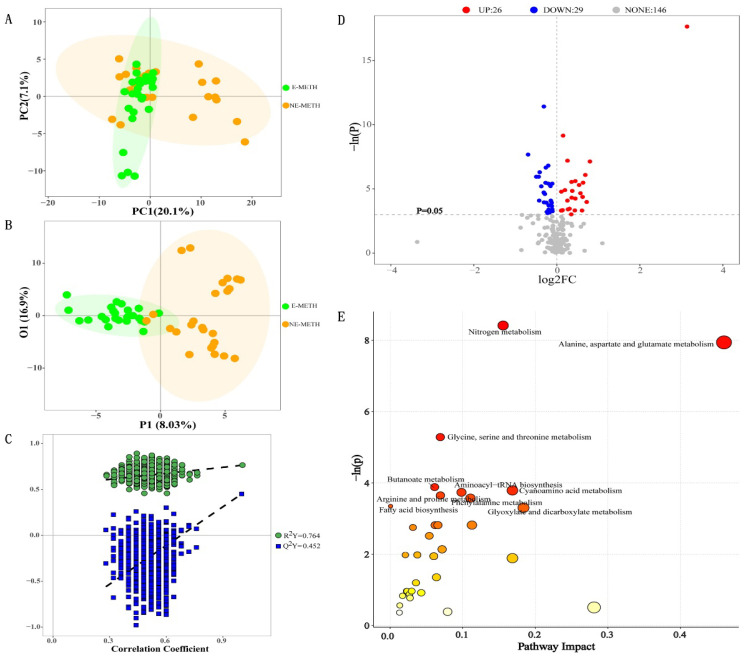
Differential metabolic profiles between the NE-METH and E-METH groups. (**A**) PCA score plot data of the NE-METH group (yellow) versus the E-METH group (green), (**B**) OPLS-DA score plot data of the NE-METH group (yellow) versus the E-METH group (green), (**C**) permutation plot of the OPLS-DA models, (**D**) volcano plot of 55 significantly altered metabolites (*p* < 0.05), and (**E**) bubble plot of altered metabolic pathways analysis obtained via KEGG analysis and the comparison of E-METH with NE-METH.

**Figure 4:**
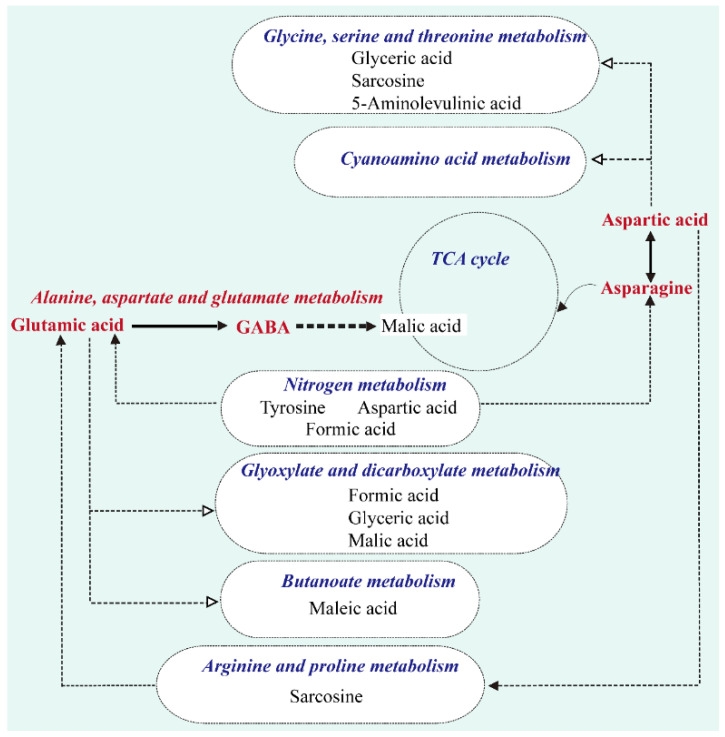
Metabolic network of significantly changed pathways and metabolites.

The PCA plots illustrated in Figure 3A revealed that NE-METH metabolic profiles were different from E-METH metabolic profiles. These results indicated significant metabolic differences between the two groups. Furthermore, the OPLS-DA model (Figure 3B) showed that the samples were divided into two clusters. The cumulative R2Y and Q2Y from the OPLS-DA score plot were 0.764 and 0.0452, respectively, and the intercept for the *Q*2 regression line was negative (Figure 3C). These results ensured the reliability of the OPLS-DA models. Then, 55 differential metabolites were selected (*p* < 0.05, Table 2). Compared with those in the NE-METH group, 26 differential metabolites (red points) were increased and 29 differential metabolites (blue points) were decreased in the E-METH group (Figure 3D). Among these differential metabolites, 51 were selected as potential biomarkers with VIP > 1 (Table 2, top 51). Then, metabolic pathway analysis was performed via KEGG. The pathways with *p* < 0.05 in the graph are labeled in Figure 3E. As shown in Figure 3E, the differential metabolites were mainly enriched in 10 pathways, including nitrogen metabolism; alanine, aspartate, and glutamate metabolism; glycine, serine, and threonine metabolism; butanoate metabolism; aminoacyl–tRNA biosynthesis; cyanoamino acid metabolism; arginine and proline metabolism; phenylalanine metabolism, fatty acid biosynthesis; and glyoxylate and dicarboxylate metabolism. Six pathways in METH addicts were significantly affected by exercise (Figure 4). These pathways were nitrogen metabolism; glycine, serine, and threonine metabolism; butanoate metabolism; cyanoamino acid metabolism; arginine and proline metabolism; and glyoxylate and dicarboxylate metabolism.

### 2.3. Comparative Changes Induced by METH and Exercise

Among the above 55 differential metabolites in Table 3, 15 demonstrated normalized trends under induction by exercise and METH. As shown in Table 3, METH reduced the levels of linoleylcarnitine, hexanylcarnitine, sebacic acid, acetylcarnitine, maleic acid, 5-aminolevulinic acid, asparagine, malic acid, and N-acetylneuraminic acid and enhanced the levels of ethylmethylacetic acid, glyceric acid, isovaleric acid, erythronic acid, glutamic acid, and threonic acid. Exercise caused the opposite changes. Additionally, 22 metabolites showed the same trends under the induction of exercise and METH (Table 4). METH and exercise reduced the levels of pentadecanoic acid, DHA, 3-methyl-2-oxopentanoic acid, adrenic acid, phenylpyruvic acid, aspartic acid, suberic acid, DPAn-6, alpha-ketoisovaleric acid, dodecanoic acid, dihomo-gamma-linolenic acid, tyrosine, oleic acid, and DPA but increased the levels of carnitine, dodecanoylcarnitine, decanoylcarnitine, formic acid, methylcysteine, glucaric acid, acetylglycine, and tetradecanoylcarnitine. Here, we listed the differential metabolites related to METH addiction (Table 5), including formic acid, tyrosine, aspartic acid, carnitine, glutamic acid, asparagine and acetylcarnitine.

Seven pathways that were most significantly affected by exercise were also induced by METH. These pathways were nitrogen metabolism; alanine, aspartate, and glutamate metabolism; glycine, serine, and threonine metabolism; butanoate metabolism; aminoacyl–tRNA biosynthesis; cyanoamino acid metabolism; and glyoxylate and dicarboxylate metabolism. Here, we listed the differential metabolic pathway related to METH addiction (Table 6).

## 3. Discussion

In addition to the stimulatory and psychotropic effects of METH on the nervous system, METH abuse drives underlying effects on biological metabolism and the turnover of peripheral transmitters [23]. Mapping metabolic disturbances to pathways can help investigate the latent pathogenesis of METH addiction, relapse, and withdrawal. The neurotoxicity of METH is thought to be related to glutamate excitotoxicity, mitochondrial dysfunction, and oxidative stress in neurons. In the present study, 201 metabolites were identified, among which the differential expression of 115 was induced by METH. These results indicated that METH significantly changed the metabolic profiles of abusers. A total of 19 pathways were identified, and glyoxylate and dicarboxylate metabolism; alanine, aspartate and glutamate metabolism; and citrate cycle (TCA cycle); and phenylalanine metabolism were the four metabolic pathways that were most affected by METH.

Glyoxylate and dicarboxylate metabolism describes a variety of reactions involving glyoxylate or dicarboxylates that interconnect with several aspects of cellular metabolism, including the TCA cycle; glycine, serine, and threonine metabolism; and nitrogen metabolism. Our data suggested that METH might stimulate glyoxylate and dicarboxylate metabolism with significantly increased levels of glyceric acid, cis-aconitic acid, citric acid, isocitric acid, glycolic acid, succinic acid, oxalic acid, and formic acid. As the end product of the glyoxylate and dicarboxylate metabolism pathway, formic acid has direct toxic effects that can cause oxidative stress, mitochondrial damage, and increased lipid peroxidation associated with the mechanism of neurotoxicity [24]. The classical mechanism of METH neurotoxicity is that METH causes oxidative stress in neurons by increasing release and reuptake blockage that then leads to excessive cytoplasmic dopamine [25]. Our results may help understand the neurochemical effects of METH and its neuroinflammatory mechanism from a metabolomic perspective.

Among the increased metabolites identified in our results, succinic acid, citric acid, cis-aconitic acid, and isocitric acid are also involved in the TCA cycle, and the stimulatory effect of METH on the TCA cycle leads to an increase in ATP supply, indicating that individuals with METH dependence require additional energy. These findings provide strong evidence for the disruption of the citric acid cycle by METH and confirm long-standing observations in this area [25,26]. Zaitsu et al. found that in drug-addicted rat models, the amounts of some metabolites, including TCA cycle intermediates, significantly changed, indicating the disruption of energy metabolism. Differences in the metabolic profiles were suggestive of the different biological states of drug addiction [27]. McClay et al. applied metabolomics to investigate the neurochemical consequences of METH exposure in the rodent brain and found that the levels of TCA metabolites, such as succinate, fumarate, and malate, were increased; these results were suggestive of an increase in energy metabolism after METH use [28]. Similarly, Zheng et al. [11] reported that the administration of METH to rats can improve energy metabolism, including accelerating the TCA cycle. Shima et al. [29] reported that METH causes energy metabolism disruption in the peripheral system. However, their results contradicted the above results. Such discrepancies could be mainly attributed to differences in sampling time points, doses, and target regions. In a study of effects on mitochondrial metabolic networks, Shima et al. [29] found that urinary levels of TCA cycle intermediates such as aconitase, alpha-ketoglutarate, malate, fumarate, succinate, oxaloacetate, pyruvate, and isocitrate/citrate were reduced in rat urine collected 0 to 24 h after the last methamphetamine injection. No differences were found in these markers compared to control rats in urine samples collected between 72 and 96 h. In addition, early studies suggested that amphetamine neurotoxicity is essentially damage to monoaminergic endpoints. Now, a growing body of evidence clearly indicates that mitochondria are the direct targets of amphetamine neurotoxicity. These events include alterations in TCA cycle enzyme function, perturbations in mitochondrial clearance mechanisms, and disturbances in mitochondrial dynamics. Moreover, amphetamine-induced neuronal toxicity is dependent on the activation of several mitochondrial pathways [30].

The metabolomics research done by previous studies on drug addiction utilized animals as the research object. Our results were based on human serum samples. Under METH exposure, energy demand increases and energy metabolism in brain regions acutely increases; these effects lead to damage to energy metabolism and monoaminergic terminals. Notably, the TCA cycle is involved in the occurrence of some neurodegenerative diseases [31,32]. Therefore, the change in the TCA cycle induced by METH may not only be related to energy metabolism but also plays an important role in the degeneration of the nervous system and the change in cognitive level in METH users. Glutamate excitotoxicity represents another mechanism through which METH causes neuronal damage. Our data showed that the alanine, aspartate, and glutamate metabolism pathway was highly significantly affected by METH. The increased serum levels of the excitatory amino acid glutamate after METH treatment were suggestive of nervous system activation and hence elevated nervous activity. We discuss this pathway combined with exercise effects below. Similar to GABA, glycine and alanine are inhibitory neurotransmitters in the brain. The markedly decreased serum levels of alanine and glycine were suggestive of reduced nervous system inhibition and elevated nervous activity and were consistent with the results of Zheng et al. [11]. Aspartic acid, as a precursor of asparagine, was observed in this work that METH caused aspartic acid to decrease the corresponding asparagine content, while exercise training reduced aspartic acid and asparagine content. Autophagy exists in METH-damaged nerves and METH alters the effectiveness of autophagosomes [33]. Asparagine, as an autophagy inhibitor, increases METH toxicity and extends DA toxicity to the mesencephalon [34].

Animal and human studies have demonstrated that exercise training effectively prevents addiction formation and suppresses drug-seeking behaviors. These observations are supported by neurobiological studies that found that exercise training modulates several neural networks, including the dopaminergic reward system, and regulates neurogenesis and spinogenesis [22]. However, intervention guidelines and biomarkers warrant further investigation. Our data identified 55 significantly different metabolites between NE-METH and E-METH groups, of which 26 were elevated and 29 were lower in the E-METH group than in the NE-METH group. The findings on these significantly exercise-varying metabolites, especially metabolites in amino acid metabolism pathways, implied that the regulation of these pathways contributes to the movement of METH abusers. Figure 4 shows the metabolic network of the significantly changed pathways and metabolites. The alanine, aspartate, and glutamate metabolism pathway was highly significantly affected by exercise. 

METH has been reported to lead to the disorder of neurotransmitters, mainly those in the glutamine–glutamate–GABA system, in peripheral blood serum/plasma [11]. Glutamate is the most important excitatory neurotransmitter in the human brain. However, METH addiction can lead to the functional imbalance of the glutamatergic system [26]. The combination of METH with the dopamine receptor increases the concentration of extracellular glutamate. The long-term over-activation of glutamatergic neurons may lead to excitotoxicity, leading to the reduction in the number of pyramidal cells and dendrites in the prefrontal cortex [35], the impairment of cognitive control function, and impulse medication. In animal experiments, studies have shown that L-carnitine levels are highly correlated with glutamatergic function and dendritic plasticity in the hippocampus [36]. Moreover, acetylcholine can cross the blood-brain barrier and act as a powerful antioxidant to protect the nervous system and benefit brain function [37]. In this work, we observed that METH caused the decrease of acetylcholine level, while the level increased after exercise training, which was consistent with the current research trend. GABA is the most important inhibitory amino acid neurotransmitter in the brain. It can be produced via the decarboxylation of glutamate. Glutamate can also balance the function of the dopamine system in the brain [38]. Therefore, glutamate homeostasis is considered to play a crucial role in the biochemical mechanism of METH addiction. Interestingly, our results showed that exercise decreased the serum levels of glutamic acid but elevated GABA, which may protect against the overstimulation of glutamatergic receptors following chronic drug exposure, increases the inhibition of the nervous system, and subsequently improves cognitive control function. These results suggested that exercise can affect drug addicts by regulating these metabolic pathways. The results of physical fitness test show that exercise can significantly improve the physical fitness of drug addicts. Vital capacity is an important index to measure cardiopulmonary function. Compared with the NE-METH group, the performance of the E-METH group was significantly improved, indicating that exercise intervention has a definite and good effect on the recovery of cardiopulmonary function in drug addicts. At the same time, exercise affects METH-induced metabolic pathway, arginine and proline metabolism are significantly affected, and this pathway is considered to be closely related to cardiopulmonary function [39]. Among the metabolites caused by METH, metabolites including glutamate and acetylcarnitine are related to the mechanism of neurotoxicity [40,41]. Fortunately, these two metabolites have been well transformed under the intervention of exercise.

## 4. Methods

### 4.1. Study Design

Fifty males with METH dependence (METH group) were recruited from the Ziyang Compulsory Isolation Drug Rehabilitation Center of Sichuan Province, P.R. China. The participants were aged 25–35 years old, and all took METH through smoking. They fulfilled the DSM-IV criteria for METH dependence [42]. Twenty-five age-matched healthy nonusers (control group) aged 25–35 years old were recruited from a community in P. R. China (Table 7). The inclusion criteria included (1) 18–45 years old; (2) compliance with the METH dependency standard in DSM-IV; (3) having education level above primary school; (4) acquired eligibility after exercise risk assessment; (5) ensuring more than half a year of rehabilitation; (6) subjects participate voluntarily and sign informed consent; (7) METH urine test showed positive in the most recent 1 year. Exclusion criteria included (1) those with infectious diseases such as hepatitis, HIV and severe trauma who are not cured; (2) recent neurological injuries such as craniocerebral injury and spinal cord injury, or severe mental diseases; (3) suffering from serious organic diseases; (4) in addition to METH dependence, there are other illicit drug dependences. The experimental procedure was approved by the Human Ethics Committee of Chengdu Sport University and was in accordance with the ethical standards specified by the Helsinki Declaration of 1975 (revised in 2008). Informed consent was obtained from all participants.

Fifty males with METH dependence were randomly divided into the NE-METH and E-METH groups. The NE-METH group did not participate in the exercise intervention, whereas the E-METH group took part in exercise intervention. Aerobic exercise intervention was carried out in the E-METH group for 48 weeks and was divided into stages I, II, and III. Real-time heart rate was recorded by using Polar Sports Tester (Polar ProTrainer 5™ SW) to control the intensity of the intervention. In stage I, the heart rate was kept at 50–60% HRmax (HRmax = 206.9 − 0.67 × age) for 4 weeks; in stage II, the heart rate was kept at 60%–70% HRmax for 10 weeks; and in stage III, the heart rate kept at 65–75% HRmax for 34 weeks. The frequency of exercise was three times a week. The subjects participated in several aerobic exercises, including jogging, jumping jacks, shuttle runs, and push-ups. All subjects completed this study, and no subjects dropped out.

Serum sample preparation for metabolic profiling was performed before and after exercise intervention, that is, the morning before the exercise intervention and the morning after the exercise, respectively. The blood samples of the NE-METH group were collected at the same time as those of the E-METH group, and the blood samples of the control group were collected at the same time as those of the E-METH group before exercise. The centrifuged serum samples were stored at −80 °C until analysis.

### 4.2. Metabolite Profiling

Metabolomic analysis was performed by using a Q300 Kit (Metabo-Profile, Shanghai, China). The samples were treated in accordance with the following steps: The samples were thawed on an ice-bath to minimize sample degradation. A total of 25 μL of serum was added to a 96-well plate. Then, the plate was transferred to an Eppendorf epMotion Workstation (Eppendorf Inc., Humburg, Germany). Each sample was automatically added with 120 μL of ice-cold methanol with partial internal standards and vortexed vigorously for 5 min. The plate was centrifuged at 4000× *g* for 30 min (Allegra X-15R, Beckman Coulter, Inc., Indianapolis, IN, USA). Subsequently, the plate was returned to the workstation. A total of 30 μL of supernatant was transferred to a clean 96-well plate, and 20 μL of freshly prepared derivative reagents was added to each well. The plate was sealed, and the derivatization was carried out at 30 °C for 60 min. After derivatization, 330 μL of ice-cold 50% methanol solution was added to dilute the sample. Then, the plate was stored at −20 °C for 20 min and then centrifuged at 4000× *g* and 4 °C for 30 min. A total of 135 μL of supernatant was transferred to a new 96-well plate with 10 μL of internal standards in each well. Serial dilutions of the derivatized stock standards were added to the remaining wells. Finally, the plate was sealed for LC–MS analysis.

An ultraperformance liquid chromatography coupled to tandem mass spectrometry (UPLC–MS/MS) system (ACQUITY UPLC-Xevo TQ-S, Waters Corp., Milford, MA, USA) was used to quantify all target metabolites. Internal standards were added to the test samples to monitor analytical variations during the entire sample preparation and analysis processes. Pooled quality control (QC) samples were prepared by mixing aliquots of the study samples such that the pooled samples broadly represented the biological average of the whole sample set. The QC samples were prepared with the test samples and injected at regular intervals (after every 14 test samples for LC–MS) throughout the analytical run.

The raw data files generated via UPLC–MS/MS were processed by using MassLynx software (v4.1, Waters, Milford, MA, USA) to perform peak integration, calibration, and quantitation for each metabolite.

### 4.3. Statistical Analysis

Statistical analysis was performed with iMAP (v1.0, Metabo-Profile, Shanghai, China) software. Principal component analysis (PCA) and orthogonal partial least squares discriminant analysis (OPLS-DA) were performed. Variable importance in projection (VIP) was identified on the basis of the OPLS-DA model. For the identification of different metabolites, Student’s t-test was used for data with a parametric distribution, and Mann–Whitney tests were used for those with a nonparametric distribution. Metabolites with *p* < 0.05 in univariate statistics were regarded as differentially expressed metabolites. Among these metabolites, the metabolites with Variable important projection (VIP) > 1 were selected as potential biomarkers. A database resource, namely, Kyoto Encyclopedia of Genes and Genomes (KEGG), was utilized to identify potentially disordered metabolic pathways (http://www.genome.jp/kegg/, accessed on 1 May 2022).

### 4.4. Reagents Setup

All of the standards of the target metabolites were obtained from Sigma–Aldrich (St. Louis, MO, USA), Steraloids Inc. (Newport, RI, USA), and TRC Chemicals (Toronto, ON, Canada). All the standards were accurately weighed and prepared in water, methanol, sodium hydroxide solution, or hydrochloric acid solution to obtain individual stock solutions with a concentration of 5.0 mg/mL. An appropriate amount of each stock solution was mixed to create stock calibration solutions.

Formic acid was of optima grade and obtained from Sigma-Aldrich (St. Louis, MO, USA). Methanol (optima LC–MS), acetonitrile (optima LC–MS), and isopropanol (optima LC–MS) were purchased from Thermo-Fisher Scientific (Fair Lawn, NJ, USA). Ultrapure water was produced by a Mill-Q Reference system equipped with a LC–MS Pak filter (Millipore, Billerica, MA, USA).

## 5. Conclusions

This study elucidated the characteristics of peripheral metabolites in METH addicts and metabolomic profiles in METH dependence following aerobic exercise training. The results showed that METH could cause continuous changes in peripheral metabolism, mainly in amino acid metabolism. However, exercise regulates the changes in metabolic profiles, and alanine, aspartate, and glutamate metabolism and nitrogen metabolism were the most affected pathways. Notably, METH increased the serum levels of glutamate and decreased GABA, whereas exercise decreased the serum levels of glutamate and increased GABA. The results suggested that METH use leads to the disturbance of metabolite profiles in METH abusers, whereas exercise could reprogram metabolic homeostasis in METH dependence.

## Figures and Tables

**Table 1:** Differential metabolites screened from the METH and control groups.

NO.	Metabolite	*p* Value	VIP	Fold Change	Variations versus Controls
1	Caproic acid	1.58 × 10^−14^	1.987	7.320	↑
2	cis-Aconitic acid	1.58 × 10^−14^	1.961	2.547	↑
3	Isovaleric acid	1.58 × 10^−14^	1.945	18.099	↑
4	Formic acid	1.58 × 10^−14^	1.913	3.164	↑
5	Pyruvic acid	1.41 × 10^−9^	1.911	0.044	↓
6	Isocitric acid	1.58 × 10^−14^	1.901	4.029	↑
7	Citric acid	2.04 × 10^−17^	1.897	2.191	↑
8	Malonic acid	1.58 × 10^−14^	1.873	3.192	↑
9	Glucaric acid	1.4 × 10^−9^	1.858	2.383	↑
10	Glutamic acid	3.16 × 10^−14^	1.854	2.192	↑
11	Pyroglutamic acid	3.16 × 10^−14^	1.847	2.436	↑
12	alpha-Ketoisovaleric acid	6.33 × 10^−14^	1.833	0.210	↓
13	Glycolic acid	1.58 × 10^−14^	1.832	3.018	↑
14	Phenylpyruvic acid	1.92 × 10^−15^	1.805	0.432	↓
15	Hydroxypropionic acid	1.58 × 10^−14^	1.779	3.491	↑
16	Oxalic acid	3.01 × 10^−13^	1.757	4.567	↑
17	Oxoglutaric acid	1.58 × 10^−14^	1.753	0.037	↓
18	Succinic acid	1.06 × 10^−12^	1.744	1.807	↑
19	Methionine	1.22 × 10^−13^	1.740	0.575	↓
20	Ketoleucine	4.3 × 10^−12^	1.724	0.499	↓
21	Butyric acid	8.38 × 10^−12^	1.714	1.975	↑
22	Xylose	1.58 × 10^−14^	1.699	0.185	↓
23	Citramalic acid	1.27 × 10^−9^	1.692	86.760	↑
24	Glutamine	8.42 × 10^−12^	1.668	0.643	↓
25	Methylmalonic acid	9.12 × 10^−11^	1.662	1.676	↑
26	Docosapentaenoic acid (DPAn-6)	7.12 × 10^−13^	1.659	0.301	↓
27	Methylcysteine	2.78 × 10^−9^	1.644	2.691	↑
28	Heptanoic acid	7.49 × 10^−11^	1.632	1.696	↑
29	3-Methyl-2-oxopentanoic acid	8.5 × 10^−10^	1.600	0.637	↓
30	Nonanoic acid	1.76 × 10^−6^	1.591	13.591	↑
31	Glucose	2.92 × 10^−10^	1.579	1.287	↑
32	Glyceric acid	8.77 × 10^−7^	1.541	2.145	↑
33	Aspartic acid	1.86 × 10^−10^	1.523	0.634	↓
34	4-Hydroxyphenylpyruvic acid	3.35 × 10^−9^	1.517	0.383	↓
35	Isocaproic acid	7.55 × 10^−9^	1.487	1.557	↑
36	Oxoadipic acid	1.43 × 10^−8^	1.470	0.708	↓
37	Malic acid	8.12 × 10^−8^	1.433	0.615	↓
38	Asparagine	5.12 × 10^−8^	1.431	0.609	↓
39	Ricinelaidic acid	9.44 × 10^−8^	1.390	0.369	↓
40	Methylsuccinic acid	1.27 × 10^−7^	1.377	1.656	↑
41	Docosahexaenoic acid (DHA)	5.81 × 10^−9^	1.373	0.511	↓
42	Threonine	5.68 × 10^−7^	1.365	0.769	↓
43	10Z-Nonadecenoic acid	3.44 × 10^−7^	1.363	0.328	↓
44	Serine	1.87 × 10^−6^	1.360	0.735	↓
45	Adrenic acid	8.12 × 10^−8^	1.357	0.565	↓
46	Glutaric acid	3.18 × 10^−8^	1.345	1.520	↑
47	Glycine	8.57 × 10^−6^	1.304	0.716	↓
48	Acetylcarnitine	1.59 × 10^−6^	1.281	0.679	↓
49	10Z-Heptadecenoic acid	3.68 × 10^−6^	1.260	0.523	↓
50	Cystine	1.83 × 10^−5^	1.255	0.647	↓
51	Maltose/Lactose	7.7 × 10^−7^	1.249	0.417	↓
52	gamma-Linolenic acid	5.98 × 10^−8^	1.223	0.543	↓
53	Oleic acid	5.6 × 10^−6^	1.222	0.613	↓
54	Cinnamic acid	2.5 × 10^−5^	1.215	0.904	↓
55	Linoleylcarnitine	7.7 × 10^−7^	1.213	0.657	↓
56	Valine	2.49 × 10^−5^	1.204	0.824	↓
57	alpha-Aminobutyric acid	4.14 × 10^−5^	1.204	0.770	↓
58	Pentadecanoic acid	1.8 × 10^−5^	1.194	0.538	↓
59	Sebacic acid	3.1 × 10^−4^	1.153	0.373	↓
60	Xylulose	5.04 × 10^−5^	1.113	0.667	↓
61	Indole-3-propionic acid	1.2 × 10^−4^	1.103	1.964	↑
62	alpha-Linolenic acid	5.98 × 10^−5^	1.096	0.632	↓
63	2-Methylpentanoic acid	1.055 × 10^−2^	1.092	0.938	↓
64	Tetradecanoylcarnitine	1.5 × 10^−4^	1.089	2.280	↑
65	Suberic acid	5.86 × 10^−3^	1.075	0.459	↓
66	N-Acetyaspartic acid	9.57 × 10^−3^	1.075	1.383	↑
67	Maleic acid	3.7 × 10^−4^	1.055	0.703	↓
68	Palmitic acid	4.3 × 10^−4^	1.053	0.693	↓
69	Mandelic acid	2.6 × 10^−4^	1.033	1.884	↑
70	N-Acetylneuraminic acid	6.85 × 10^−6^	1.025	0.721	↓
71	Propionylcarnitine	1.7 × 10^−4^	1.023	0.778	↓
72	Indoleacrylic acid	3.8 × 10^−4^	1.022	1.965	↑
73	Dodecanoylcarnitine	1.86 × 10^−3^	0.989	1.355	↑
74	Adipoylcarnitine	4.7 × 10^−4^	0.981	1.192	↑
75	Docosapentaenoic acid (DPA)	1.86 × 10^−3^	0.981	0.704	↓
76	Histidine	8.49 × 10^−3^	0.979	0.941	↓
77	Threonic acid	1.88 × 10^−3^	0.915	1.410	↑
78	2-Hydroxybutyric acid	7 × 10^−4^	0.913	0.730	↓
79	Erythronic acid	2.11 × 10^−3^	0.906	1.423	↑
80	Adipic acid	5.03 × 10^−3^	0.887	2.028	↑
81	Pyrrole-2-carboxylic acid	3.81 × 10^−3^	0.877	1.164	↑
82	Decanoylcarnitine	3.72 × 10^−3^	0.870	1.649	↑
83	Creatine	9.01 × 10^−3^	0.856	0.739	↓
84	Lysine	3.59 × 10^−3^	0.845	1.180	↑
85	Phenylalanine	1.282 × 10^−2^	0.826	0.760	↓
86	Nicotinic acid	1.5 × 10^−3^	0.826	1.375	↑
87	Valeric acid	1.71 × 10^−3^	0.825	2.099	↑
88	Tyrosine	8.22 × 10^−3^	0.810	0.890	↓
89	Indole-3-methyl acetate	4.1 × 10^−4^	0.781	10.018	↑
90	Isovalerylcarnitine	4.943 × 10^−2^	0.777	0.708	↓
91	bHDCA	1.142 × 10^−2^	0.751	0.627	↓
92	Lithocholic acid (LCA)	3.3 × 10^−4^	0.749	2.182	↑
93	Acetylglycine	8.49 × 10^−3^	0.738	1.116	↑
94	Homocitrulline	2 × 10^−2^	0.730	0.827	↓
95	Dodecanoic acid	1.231 × 10^−2^	0.727	0.635	↓
96	Linoleic acid	1.038 × 10^−2^	0.726	0.758	↓
97	Glycohyodeoxycholic acid (GHDCA)	2.58 × 10^−3^	0.724	0.904	↓
98	Fructose	3.06 × 10^−5^	0.711	0.604	↓
99	Dihomo-gamma-linolenic acid	3.25 × 10^−3^	0.694	0.716	↓
100	Fumaric acid	1.747 × 10^−2^	0.683	2.498	↑
101	Glycylproline	3.39 × 10^−5^	0.669	1.255	↑
102	Gluconolactone	2.226 × 10^−2^	0.662	1.211	↑
103	Tricarboxylic acid (TCA)	2.146 × 10^−2^	0.646	0.518	↓
104	beta-Alanine	2 × 10^−3^	0.636	0.701	↓
105	Shikimic acid	2.84 × 10^−3^	0.636	3.360	↑
106	Myristic acid	3.021 × 10^−2^	0.631	0.734	↓
107	Carnitine	3.477 × 10^−2^	0.591	1.142	↑
108	3-Hydroxylisovalerylcarnitine	3.36 × 10^−2^	0.583	0.918	↓
109	9E-tetradecenoic acid	1.436 × 10^−2^	0.568	1.299	↑
110	5-Aminolevulinic acid	3.531 × 10^−2^	0.545	0.872	↓
111	Ursodeoxycholic Acid (UDCA)	1.07 × 10^−3^	0.509	1.983	↑
112	2-Phenylpropionate	3.196 × 10^−2^	0.399	1.839	↑
113	Hexanylcarnitine	2.47 × 10^−3^	0.315	0.678	↓
114	Ethylmethylacetic acid	1.5 × 10^−8^	0.306	175.496	↑
115	Indole-3-carboxylic acid	1.641 × 10^−2^	0.097	401.533	↑

↑: the metabolite is elevated compared to the Control group; ↓: the metabolite is decreased compared to the Control group.

**Table 2:** Differential metabolites screened from the E-METH and NE-METH groups.

NO.	Metabolite	*p*-Value	VIP	Fold Change	Variations versus NE-METH
1	Benzoic acid	2.2 × 10^−8^	2.424	8.722	↓
2	Linoleylcarnitine	1.12 × 10^−5^	2.450	0.797	↑
3	Ethylmethylacetic acid	1.1 × 10^−4^	1.388	1.100	↓
4	Hexanylcarnitine	4.7 × 10^−4^	1.970	0.613	↑
5	Glyceric acid	7.6 × 10^−4^	1.574	1.184	↓
6	Pentadecanoic acid	8.1 × 10^−4^	1.682	1.722	↓
7	Carnitine	1.11 × 10^−3^	1.982	0.858	↑
8	3-Hydroxyisovaleric acid	1.3 × 10^−3^	2.024	0.824	↑
9	Octanoylcarnitine	1.86 × 10^−3^	1.711	0.743	↑
10	Isovaleric acid	2.3 × 10^−3^	1.471	1.594	↓
11	Sebacic acid	2.65 × 10^−3^	1.152	0.703	↑
12	Dodecanoylcarnitine	2.65 × 10^−3^	1.962	0.731	↑
13	DHA	3.72 × 10^−3^	1.655	1.349	↓
14	3-Methyl-2-oxopentanoic acid	3.97 × 10^−3^	1.712	1.267	↓
15	Arachidonic acid	4.21 × 10^−3^	1.648	1.536	↓
16	Decanoylcarnitine	4.24 × 10^−3^	1.994	0.820	↑
17	Acetylcarnitine	4.53 × 10^−3^	1.545	0.859	↑
18	Glutarylcarnitine	4.53 × 10^−3^	1.595	0.916	↑
19	Adrenic acid	5.05 × 10^−3^	1.576	1.437	↓
20	Formic acid	5.5 × 10^−3^	2.075	0.891	↑
21	Isobutyric acid	5.6 × 10^−3^	1.530	0.764	↑
22	Phenylpyruvic acid	7.51 × 10^−3^	1.632	1.134	↓
23	Aspartic acid	7.99 × 10^−3^	1.377	1.281	↓
24	Suberic acid	8.49 × 10^−3^	1.737	1.068	↓
25	2-Hydroxy-3-methylbutyric acid	9.01 × 10^−3^	1.789	0.797	↑
26	Erythronic acid	9.57 × 10^−3^	1.426	1.470	↓
27	alpha-Hydroxyisobutyric acid	9.86 × 10^−3^	1.567	0.807	↑
28	Maleic acid	1.015 × 10^−2^	1.702	0.811	↑
29	Palmitelaidic acid	1.275 × 10^−2^	1.290	1.522	↓
30	Stearylcarnitine	1.357 × 10^−2^	1.074	1.270	↓
31	DPAn-6	1.447 × 10^−2^	1.541	1.349	↓
32	1H-Indole-3-acetamide	1.698 × 10^−2^	1.125	0.738	↑
33	alpha-Ketoisovaleric acid	1.698 × 10^−2^	1.303	1.182	↓
34	Dodecanoic acid	1.895 × 10^−2^	1.120	1.631	↓
35	Methylcysteine	1.945 × 10^−2^	1.512	0.800	↑
36	Gamma-aminobutyric acid (GABA)	2 × 10^−2^	1.425	0.908	↑
37	5-Aminolevulinic acid	2 × 10^−2^	1.645	0.832	↑
38	Sarcosine	2.474 × 10^−2^	1.368	0.865	↑
39	Glucaric acid	2.606 × 10^−2^	1.475	0.911	↑
40	Dihomo-gamma-linolenic acid	3.203 × 10^−2^	1.256	1.224	↓
41	Octanoic acid	3.36 × 10^−2^	1.572	0.916	↑
42	eicosapentaenoic acid (EPA)	3.36 × 10^−2^	1.245	1.193	↓
43	Acetylglycine	3.408 × 10^−2^	1.514	0.864	↑
44	Tyrosine	3.569 × 10^−2^	1.195	1.090	↓
45	Oleic acid	3.652 × 10^−2^	1.210	1.338	↓
46	DPA	3.677 × 10^−2^	1.133	1.508	↓
47	Glutamic acid	3.709 × 10^−2^	1.394	1.078	↓
48	Asparagine	3.895 × 10^−2^	1.459	0.920	↑
49	Tetradecanoylcarnitine	4.289 × 10^−2^	1.600	0.877	↑
50	Malic acid	4.498 × 10^−2^	1.273	0.849	↑
51	Threonic acid	4.943 × 10^−2^	1.334	1.260	↓
52	Phthalic acid	4.289 × 10^−2^	0.935	0.871	↑
53	N-Acetylneuraminic acid	4.088 × 10^−2^	0.850	0.838	↑
54	Isoleucine	3.709 × 10^−2^	0.764	1.064	↓
55	Ornithine	1.698 × 10^−2^	0.755	0.895	↑

↑: the metabolite is elevated compared to the NE-METH group; ↓: the metabolite is decreased compared to the NE-METH group.

**Table 3:** Metabolites with different trends under exercise and METH.

NO.	Metabolite	Variations with Exercise	Variations with METH
1	Linoleylcarnitine	↑	↓
2	Hexanylcarnitine	↑	↓
3	Sebacic acid	↑	↓
4	Acetylcarnitine	↑	↓
5	Maleic acid	↑	↓
6	5-Aminolevulinic acid	↑	↓
7	Asparagine	↑	↓
8	Malic acid	↑	↓
9	N-Acetylneuraminic acid	↑	↓
10	Ethylmethylacetic acid	↓	↑
11	Glyceric acid	↓	↑
12	Isovaleric acid	↓	↑
13	Erythronic acid	↓	↑
14	Glutamic acid	↓	↑
15	Threonic acid	↓	↑

↑: the metabolite is elevated compared to the NE-METH group; ↓: the metabolite is decreased compared to the NE-METH group;↑: the metabolite is elevated compared to the Control group; ↓: the metabolite is decreased compared to the Control group.

**Table 4:** Metabolites with same trends under exercise and METH.

NO.	Metabolite	Variations with Exercise	Variations with METH
1	Carnitine	↑	↑
2	Dodecanoylcarnitine	↑	↑
3	Decanoylcarnitine	↑	↑
4	Formic acid	↑	↑
5	Methylcysteine	↑	↑
6	Glucaric acid	↑	↑
7	Acetylglycine	↑	↑
8	Tetradecanoylcarnitine	↑	↑
9	Pentadecanoic acid	↓	↓
10	DHA	↓	↓
11	3-Methyl-2-oxopentanoic acid	↓	↓
12	Adrenic acid	↓	↓
13	Phenylpyruvic acid	↓	↓
14	Aspartic acid	↓	↓
15	Suberic acid	↓	↓
16	DPAn-6	↓	↓
17	alpha-Ketoisovaleric acid	↓	↓
18	Dodecanoic acid	↓	↓
19	Dihomo-gamma-linolenic acid	↓	↓
20	Tyrosine	↓	↓
21	Oleic acid	↓	↓
22	DPA	↓	↓

↑: the metabolite is elevated compared to the NE-METH group; ↓: the metabolite is decreased compared to the NE-METH group; ↑: the metabolite is elevated compared to the Control group; ↓: the metabolite is decreased compared to the Control group.

**Table 5:** METH-related differential metabolites.

NO.	Metabolite
1	Formic acid
2	Tyrosine
3	Aspartic acid
4	Carnitine
5	Glutamic acid
6	Asparagine
7	Acetylcarnitine

**Table 6:** METH-related differential metabolic pathways.

NO.	Pathway
1	glyoxylate and dicarboxylate metabolism
2	alanine, aspartate and glutamate metabolism
3	TCA cycle
4	phenylalanine metabolism

**Table 7:** Basic information of the experimental subjects.

Characteristics	Control Group (n = 25)	NE-METH Group (n = 25)	E-METH Group (n = 25)
Age (years)	29.20 ± 2.60	29.0 ± 4.93	28.7 ± 4.49
Height (cm)	168.31 ± 6.19	168.85 ± 5.08	168.89 ± 4.85
Weight (kg)	69.24 ± 8.43	69.07 ± 8.38	69.5 ± 7.31
Duration of continuous METH use (months)	/	61.10 ± 9.10	62.56 ± 1.454

## Data Availability

The data presented in this study are available within the article. Patients’ information are restricted due to privacy and ethical considerations.

## Supplementary Materials

The following supporting information can be downloaded at: https://www.mdpi.com/article/10.3390/metabo12070606/s1, Table S1: Metabolite levels in the serum of each group.
